# A Manganese-independent Aldolase Enables Staphylococcus aureus To Resist Host-imposed Metal Starvation

**DOI:** 10.1128/mbio.03223-22

**Published:** 2023-01-04

**Authors:** Paola K. Párraga Solórzano, Talina S. Bastille, Jana N. Radin, Thomas E. Kehl-Fie

**Affiliations:** a Department of Microbiology, University of Illinois Urbana-Champaign, Urbana, Illinois, USA; b Carl R. Woese Institute for Genomic Biology, University of Illinois Urbana-Champaign, Urbana, Illinois, USA; Mississippi State University

**Keywords:** *Staphylococcus aureus*, aldolase, nutritional immunity, calprotectin, manganese

## Abstract

The preferred carbon source of Staphylococcus aureus and many other pathogens is glucose, and its consumption is critical during infection. However, glucose utilization increases the cellular demand for manganese, a nutrient sequestered by the host as a defense against invading pathogens. Therefore, bacteria must balance glucose metabolism with the increasing demand that metal-dependent processes, such as glycolysis, impose upon the cell. A critical regulator that enables S. aureus to resist nutritional immunity is the ArlRS two-component system. This work revealed that ArlRS regulates the expression of FdaB, a metal-independent fructose 1,6-bisphosphate aldolase. Further investigation revealed that when S. aureus is metal-starved by the host, FdaB functionally replaces the metal-dependent isozyme FbaA, thereby allowing S. aureus to resist host-imposed metal starvation in culture. Although metal-dependent aldolases are canonically zinc-dependent, this work uncovered that FbaA requires manganese for activity and that FdaB protects S. aureus from manganese starvation. Both FbaA and FdaB contribute to the ability of S. aureus to cause invasive disease in wild-type mice. However, the virulence defect of a strain lacking FdaB was reversed in calprotectin-deficient mice, which have defects in manganese sequestration, indicating that this isozyme contributes to the ability of this pathogen to overcome manganese limitation during infection. Cumulatively, these observations suggest that the expression of the metal-independent aldolase FdaB allows S. aureus to alleviate the increased demand for manganese that glucose consumption imposes, and highlights the cofactor flexibility of even established metalloenzyme families.

## INTRODUCTION

For many bacterial pathogens, including Staphylococcus aureus, glucose is the preferred carbon source for growth, with reductions in the ability to consume glucose ablating virulence (1–9). The value of glucose consumption to invaders is highlighted by the increased risk of infection with S. aureus, Escherichia coli, Streptococcus pneumoniae, Mycobacterium tuberculosis, Klebsiella pneumoniae, and Candida albicans that is faced by diabetic and hyperglycemic individuals (10–12). While glucose consumption is necessary for and enhances infections, many glycolytic enzymes require metals, such as manganese (Mn) and zinc (Zn), to function, and this increases the cellular demand for these essential nutrients (2, 3, 13–19). This creates a conflict that pathogens must resolve as the host restricts the availability of metals during infection (20, 21). Thus, bacteria must balance their need to consume glucose with the increased demand for metals that glycolysis places upon the cell. How S. aureus and other pathogens balance these competing demands remains largely unknown.

Glycolysis increases the staphylococcal demand for Mn (13, 22, 23). In a nutrient-replete environment, the increased cellular demand for metals that glycolysis places on the cell does not pose a challenge. However, during infection, essential metals, including Mn, iron (Fe), and Zn, are withheld from invaders by the host (24–28). The impact of this defense, known as nutritional immunity, is far-reaching, with 50% of enzymes and 30% of proteins being predicted to require a metal cofactor for proper function (29, 30). Differing from organic molecules that can frequently be synthesized as needed, inorganic nutrients, such as transition metals, must be obtained from the environment. As a result, nutritional immunity disrupts a wide range of cellular processes in pathogens, including those involved in metabolism and virulence (13, 14, 27, 31–34). A critical component of the host’s nutrient withholding response is the immune effector calprotectin (CP) (21, 26, 35). The loss of CP ablates the ability of the host to restrict metals during infection and renders mice more susceptible to disease by multiple bacterial and fungal pathogens, including S. aureus, A. baumannii, and K. pneumoniae (23, 26, 28, 36–38). CP is the most abundant protein in the cytosol of neutrophils, and its concentration can exceed 1 mg/mL in areas of infection (39, 40). CP is a heterodimer of S100A8 and S100A9 and possesses two metal-binding sites that can tightly bind Mn, Zn, and other transition metals (41–45). In addition to inhibiting processes that are necessary for optimal growth, such as glycolysis, CP-imposed metal starvation also inhibits the activity of enzymes that are essential for bacteria to survive the onslaught of the immune response, such as Mn-dependent superoxide dismutases (SODs) (13, 27).

To successfully overcome nutritional immunity, pathogens rely on the expression of high-affinity metal uptake systems and adaptation (46). S. aureus, for instance, expresses two Mn transporters: MntH, a natural resistance-associated macrophage protein (NRAMP) family member, and MntABC, an ATP-binding cassette (ABC) permease (22, 23, 47). The adaptations that facilitate success in the face of nutritional immunity include the reduced utilization of metal-dependent processes and the activation of alternative pathways and enzymes that do not rely on the restricted metal (13, 48–55). In addition to glucose, S. aureus can use amino acids as an energy source and can thus reduce its cellular demand for Mn (13). However, S. aureus must retain the ability to consume glucose to cause infection (2, 56). S. aureus and other bacteria frequently coordinate their responses to metal limitation by using the metal-sensing regulators Fur (Fe), Zur (Zn), and MntR (Mn) (46, 47, 57, 58). However, other regulatory systems that do not directly sense metal availability, such as the staphylococcal two-component system (TCS) ArlRS, also contribute to overcoming Mn starvation and nutritional immunity (14). ArlRS directly and indirectly controls the expression of numerous virulence determinants, including toxins, exoenzymes, immune modulators, and cell surface proteins involved in clumping and adherence (59–65). This TCS appears to sense the alterations in metabolic flux that occur in the latter half of glycolysis, which can be caused by Mn limitation and elevated pyruvate concentration (14, 62). Upon activation, ArlRS facilitates a metabolic shift toward amino acid utilization, thereby reducing the cellular demand for Mn (13). Consistent with its expansive regulatory network, the loss of ArlRS reduces the ability of S. aureus to cause disease in several animal models of infection (13, 61, 65–67). Notably, when the host cannot sequester Mn, the need for ArlRS during infection is ablated (13). Cumulatively, this indicates that ArlRS significantly contributes to coordinating the staphylococcal metabolic response to Mn limitation during infection.

The current studies seek to better understand how ArlRS contributes to coordinating the metabolic response of S. aureus to nutritional immunity. This work reveals that this TCS controls the expression of a metal-independent variant of aldolase (FdaB) that functionally replaces a metal-dependent isozyme (FbaA) when S. aureus is metal-starved by the host. While metal-dependent aldolases classically rely on Zn for activity, further investigation revealed that FbaA utilizes Mn and that FdaB enables S. aureus to survive host-imposed Mn limitation.

## RESULTS

### Gene regulation upon ArlRS activation.

**FIG 1:**
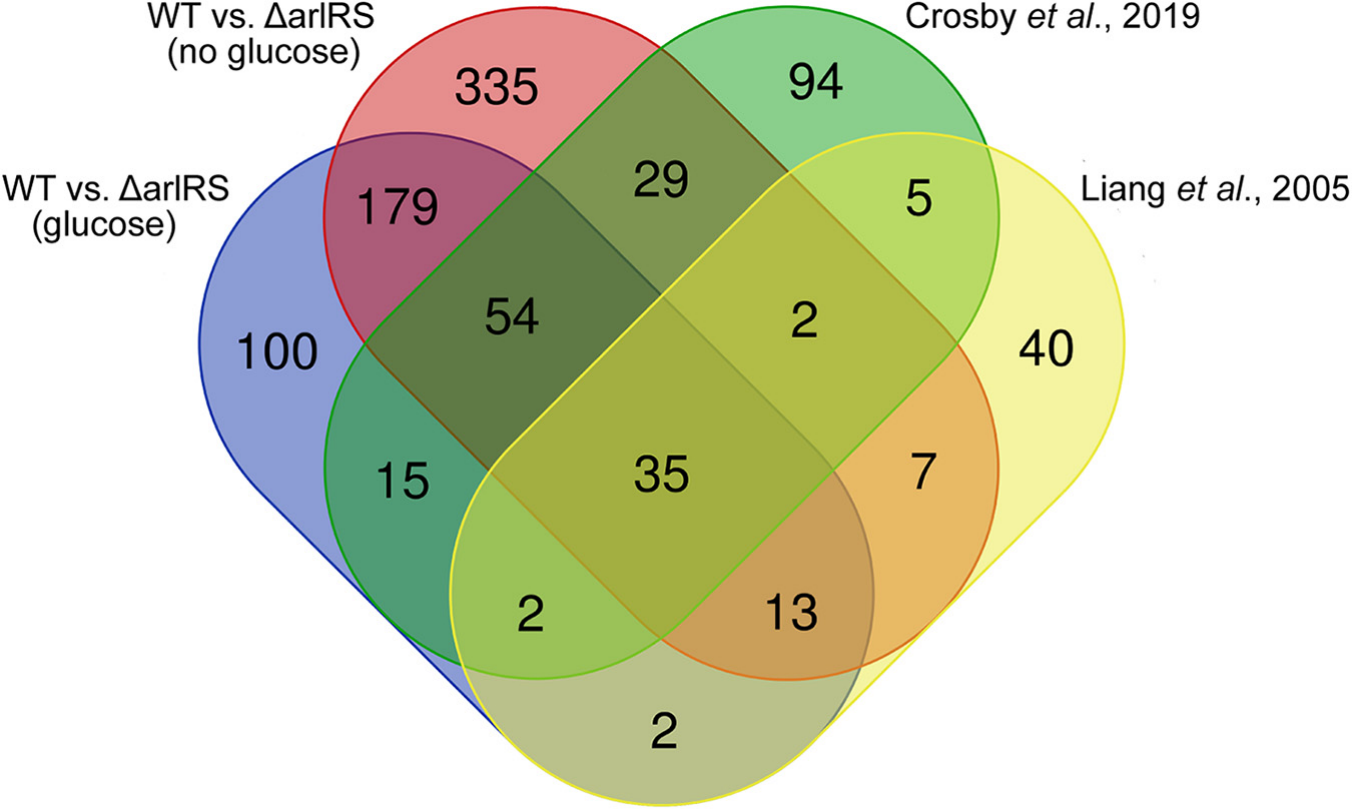
Gene regulation upon ArlRS activation. Venn diagram depicting the genes regulated by ArlRS in the absence and presence of glucose and overlap with ArlRS regulons obtained in previous studies (60, 64).

Two prior studies have elucidated the ArlRS regulon (60, 64). However, these studies were conducted prior to the identification of the signals that activate ArlRS (14, 62). Thus, these studies only compared gene expression differences between wild-type bacteria and strains lacking the ArlRS system in standard culture conditions. Both prior studies compared gene expression between the wild-type and a strain lacking a functional ArlRS system, following growth in TBS to mid-exponential phase, with Liang et al. harvesting RNA from WCUH29 (OD_600_ of 0.4) and Crosby et al. harvesting RNA from USA300 LAC (OD_600_ of 1.5). The absence of glucose was recently observed to activate ArlRS (14, 68). This finding was leveraged to better understand the ArlRS regulon by comparing gene expression between wild-type bacteria and Δ*arlRS*, following growth in the presence and absence of glucose. The use of glucose limitation as an activating signal allows for the activation of ArlRS while minimizing of the impact of the activating stimulus on the growth of the wild-type bacteria and Δ*arlRS*. This analysis, which harvested RNA from exponentially growing S. aureus Newman at an OD_600_ of 0.1, revealed that, in the presence of glucose, 183 genes are downregulated and 217 are upregulated in the Δ*arlRS* mutant, compared to the wild-type S. aureus Newman (Table S1). The apparent activity in the absence of an activating signal is consistent with the prior observation that even in the absence of ArlS, ArlR can drive the expression of the *mgrA* P2 promoter (68). In the absence of glucose, when ArlS is active, 202 genes are downregulated and 452 are upregulated in Δ*arlRS*, compared to the wild-type strain (Table S2). In total, using a 2-fold change in transcript abundance, 614 genes whose expression is modulated by ArlRS were unique to the current analysis (Fig. 1). Notably, despite this increased number of ArlRS-regulated genes in the current data set, it did not fully encompass all of the genes identified by prior analyses (60, 64). 39 genes were shared between all three studies, whereas 137 genes were shared when only data sets generated using RNA-seq were considered (Table S3) (60, 64). Prior work observed that the genes whose expression is influenced by ArlRS are dependent on the specific activating stimulus (69). Thus, it seems likely that the difference between the current work and previous work can be attributed to a combination of assaying gene expression in the presence and absence of an activating signal, differences in culture medium, and the growth phase, with the shared genes perhaps representing a core regulon. Regardless, the current analysis further establishes ArlRS as a significant modulator of staphylococcal gene expression.

10.1128/mbio.03223-22.1 TABLE S1 List of genes whose expression changes between the wild-type and Δ*arlRS*, following growth in glucose-containing medium. Download Table S1, XLSX file, 0.04 MB. Copyright © 2023 Párraga Solórzano et al. 2023 Párraga Solórzano et al. https://creativecommons.org/licenses/by/4.0/ This content is distributed under the terms of the Creative Commons Attribution 4.0 International license.

10.1128/mbio.03223-22.2 TABLE S2 List of genes whose expression changes between the wild-type and Δ*arlRS*, following growth in medium without glucose. Download Table S2, XLSX file, 0.1 MB. Copyright © 2023 Párraga Solórzano et al. 2023 Párraga Solórzano et al. https://creativecommons.org/licenses/by/4.0/ This content is distributed under the terms of the Creative Commons Attribution 4.0 International license.

10.1128/mbio.03223-22.3 TABLE S3 List of common changes between wild-type S. aureus and strains lacking ArlRS from this study and from Crosby et al. Download Table S3, XLSX file, 0.01 MB. Copyright © 2023 Párraga Solórzano et al. 2023 Párraga Solórzano et al. https://creativecommons.org/licenses/by/4.0/ This content is distributed under the terms of the Creative Commons Attribution 4.0 International license.

### FdaB promotes resistance to calprotectin.

**FIG 2:**
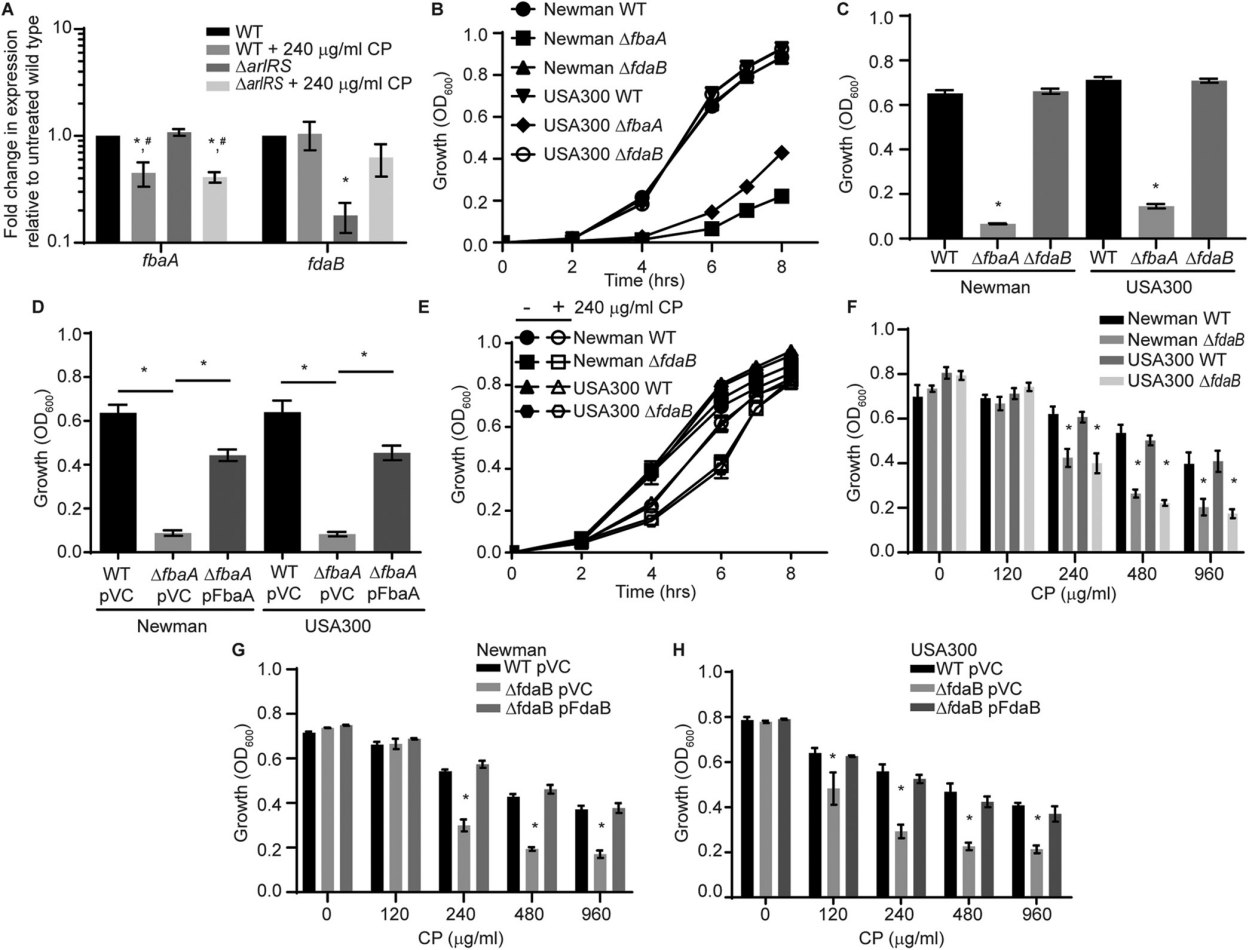
FdaB promotes resistance to calprotectin. (A) Wild-type S. aureus Newman was grown in TSB with glucose in the presence and absence of 240 μg/mL of CP, and the transcript levels of *fbaA* and *fdaB* were assessed via qRT-PCR. *, *P* ≤ 0.05 relative to the wild-type by a two-way ANOVA with Tukey’s multiple-comparison test. #, *P* ≤ 0.05 relative to the same strain in the absence of CP by a two-way ANOVA with Tukey’s multiple-comparison test. (B and C) S. aureus Newman, USA300 JE2 wild-type, Δ*fbaA*, and Δ*fdaB* were grown overnight in TSB, subcultured 1:100 in medium containing 38% TSB and 62% CP buffer, and grown in the absence of calprotectin. Growth was assessed via the measurement of the optical density, with panel C showing the OD_600_ values at t = 6 h. *, *P* ≤ 0.05 relative to the wild-type by a one-way ANOVA with Tukey’s multiple-comparison test. (D) Wild-type bacteria and Δ*fbaA* containing either an empty pOS1 plgt (pVC) or pOS1 plgt:*fbaA* (pFbaA) were grown as described in panels B and C. Growth was assessed at 6 h via the measurement of the optical densitiy. *, *P* ≤ 0.05 for the indicated comparison by a one-way ANOVA with Sidak’s multiple-comparison test. (E and F) S. aureus Newman, USA300 wild-type, Δ*fdaB*, and (G and H) S. aureus wild-type and Δ*fdaB* pOS1 plgt (pVC) or pOS1 plgt:*fdaB* (pFdaB) were assessed for CP sensitivity following growth in NRPMI containing 1 μM Zn, 1 μM Mn, and 1 μM Fe. Growth was assessed via the measurement of the absorbance at OD_600_ (F, G, and H) at t = 6 h. *, *P* ≤ 0.05 relative to the wild-type by a two-way ANOVA with Tukey’s multiple-comparison test. *n* ≥ 3. Error bars indicate the SEM.

Among the genes regulated by ArlRS is *fdaB*, whose expression decreases 4-fold in Δ*arlRS* when glucose is present. This result is similar to previous observations by Crosby et al. (64) and was confirmed using qRT-PCR (Fig. 2A). FdaB is a fructose 1,6-bisphosphate aldolase that does not require a metal ion for activity and has previously been implicated in the facilitation of gluconeogenesis (70). S. aureus also possesses a putatively Zn-dependent aldolase, FbaA (71), which is associated with glycolytic flux. During glycolysis, aldolase converts fructose 1,6-bisphosphate into glyceraldehyde 3-phosphate and dihydroxyacetone 3-phosphate, and it performs the reverse reaction during gluconeogenesis. Given this prior connection to glycolytic flux, the individual contributions of the aldolases to S. aureus growth were evaluated in a TSB-based, metal-replete, glucose-containing medium. While the loss of FdaB did not affect the ability of S. aureus to grow under these conditions, a Δ*fbaA* mutation resulted in a pronounced growth defect in both strain Newman and the USA300 strain JE2 (Fig. 2B and C). The ectopic expression of FbaA from a plasmid reversed the growth defect in both strains (Fig. 2D). These findings are consistent with potential unique roles in glycolysis and/or gluconeogenesis, and they suggest that FbaA is necessary in metal-replete environments. However, the presence of metal-dependent and independent variants of aldolase is reminiscent of the Mn-dependent and Mn-independent phosphoglycerate mutase (PGM) isozymes that are possessed by S. aureus and Salmonella enterica Typhimurium. Whereas the PGM isozymes were previously thought to contribute to glycolysis and gluconeogenesis, respectively, the Mn-independent variants of PGM enable both of these pathogens to maintain glycolytic flux when Mn-starved by the host (72). Therefore, the contribution of FdaB to resistaning host-imposed metal starvation was evaluated. For these and subsequent assays with CP, an NRPMI-based medium was used. This medium contains glucose and was supplemented with Mn, Zn, and Fe such that it would be metal-replete in the absence of CP. The loss of FdaB reduced the ability of S. aureus Newman and USA300 JE2 to grow in the presence of CP (Fig. 2E and F). The constitutive expression of FdaB from a plasmid reversed the phenotype (Fig. 2G and H). Overall, these observations indicate that FdaB is necessary for S. aureus to resist CP-imposed metal starvation.

### ArlRS is not needed to induce FdaB in response to host-imposed metal starvation.

**FIG 3:**
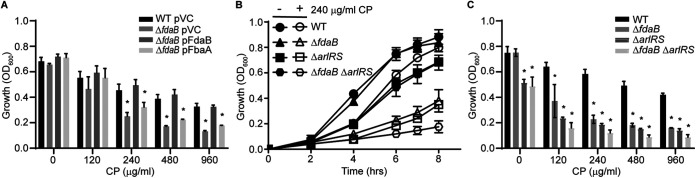
ArlRS is not needed to induce FdaB in response to host-imposed metal starvation. (A) S. aureus Newman wild-type, Δ*fdaB* containing pOS1 plgt (pVC), pOS1 plgt:*fdaB* (pFdaB), or pOS1 plgt:*fbaA* (pFbaA), and (B and C) Newman wild-type and Δ*fdaB*, Δ*arlRS*, or Δ*fdaB*Δ*arlRS* were assessed for CP sensitivity in NRPMI containing 1 μM Zn, 1 μM Mn, and 1 μM Fe. Growth was assessed via the measurement of the absorbance at OD_600_ at (A and C) t = 6 h. (A) *, *P* ≤ 0.05 relative to the wild-type containing an empty vector control at the same CP concentration by a two-way ANOVA with Tukey’s multiple-comparison test. (C) *, *P* ≤ 0.05 relative to the wild-type at the same CP concentration by a two-way ANOVA with Tukey's multiple-comparison test. *n* ≥ 3. Error bars indicate the SEM.

Having established that FdaB contributes to the resistance against metal starvation, the necessity of ArlRS for FdaB expression in the presence of CP was also assessed. In the presence of CP, a similar level of transcript was observed in Δ*arlRS*, compared to wild-type bacteria (Fig. 2A). This suggests the existence of another regulator that can induce the expression of *fdaB* in a metal-deplete medium. The expression of *fbaA* was also assessed and was observed to decrease in response to CP in both the wild-type bacteria and in a Δ*arlRS* mutant (Fig. 2A). This observation raised the possibility that the need for FdaB is due to the reduced expression of FbaA in the presence of CP. However, the constitutive expression of FbaA from a plasmid does not rescue the growth of Δ*fdaB* in metal-deplete medium (Fig. 3A). This suggests that the need for FdaB is not simply due to the reduced expression of FbaA. The sensitivity of *ΔfdaB*, Δ*arlRS*, and Δ*arlRS*Δ*fdaB* to CP was also assessed. While both *ΔfdaB* and Δ*arlRS* were more sensitive to CP than were the wild-type bacteria, the growth defect upon the loss of ArlRS was more pronounced than the loss of FdaB (Fig. 3B and C). However, the growth of Δ*arlRS*Δ*fdaB* was not further impaired, compared to the Δ*arlRS* mutant (Fig. 3B and C). Cumulatively, these results suggest that ArlRS induces the expression of *fdaB*. However, this TCS is not necessary to express the metal-independent aldolase isozyme in response to CP.

### FdaB facilitates growth in manganese-limited environments.

**FIG 4:**
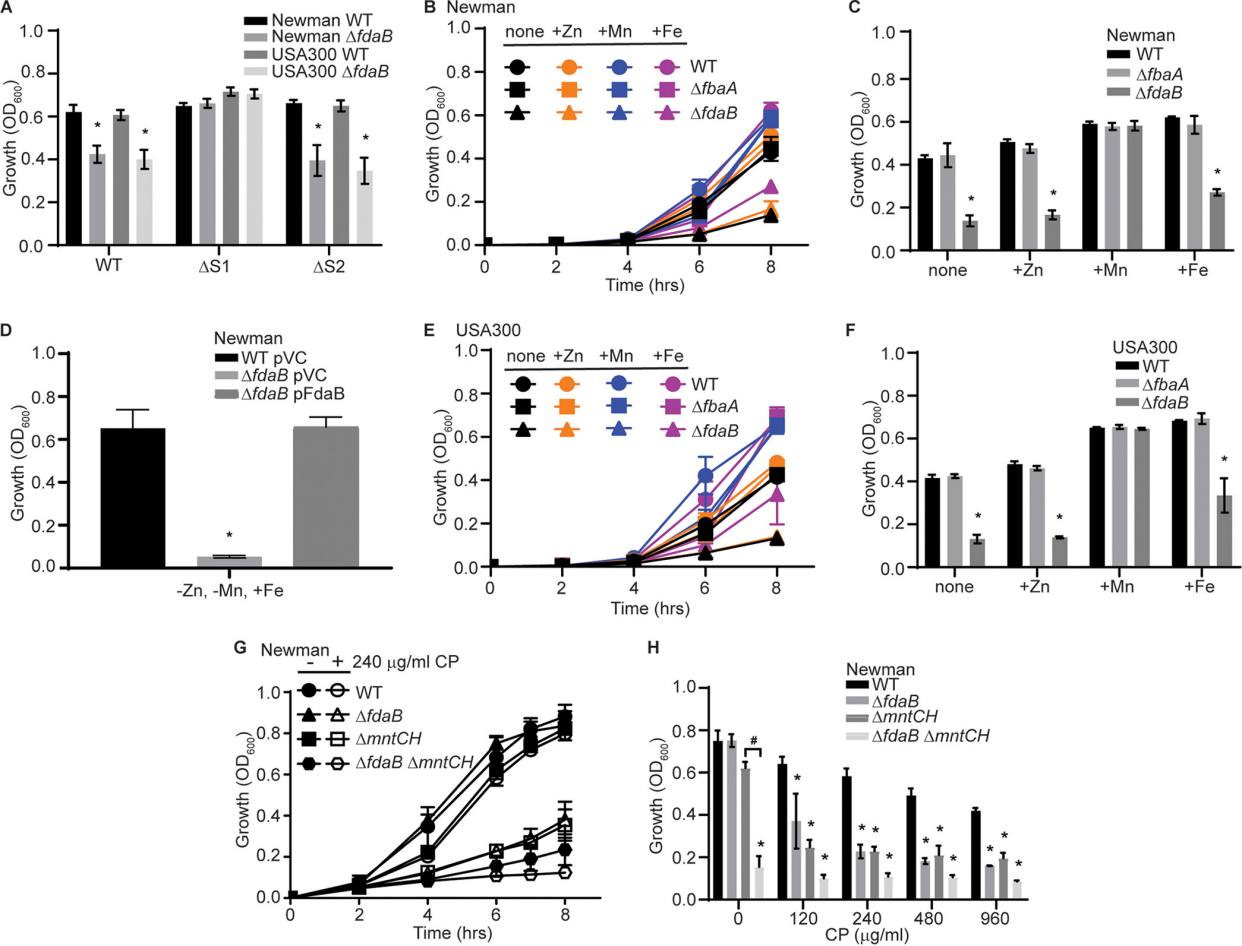
FdaB facilitates growth in manganese-limited environments. (A) S. aureus Newman and USA300 (JE2) wild-type and Δ*fdaB* mutants were assessed for sensitivity to 240 μg/mL of wild-type CP or the ΔS1 and ΔS2 variants following growth in NRPMI containing 1 μM Zn, 1 μM Mn, and 1 μM Fe. Growth was assessed via the measurement of the absorbance at OD_600_ at t = 6 h. *, *P* ≤ 0.05 relative to the wild-type with the same CP variant by a two-way ANOVA with Tukey’s multiple-comparison test. (B and C) S. aureus Newman wild-type, Δ*fbaA*, Δ*fdaB*, as well as (D) Newman wild-type and Δ*fdaB* containing pOS1 plgt (pVC) or pOS1 plgt:*fdaB* (pFdaB) and (E and F) USA300 JE2 wild-type, Δ*fbaA* and Δ*fdaB* were grown in NRPMI containing 1 μM of the indicated metal. Growth was assessed via the measurement of the absorbance at OD_600_ (C, D, and F) at t = 8 h. (C and F) *, *P* ≤ 0.05 relative to the wild-type at the same growth condition by a two-way ANOVA with Tukey’s multiple-comparison test. (D) *, *P* ≤ 0.05 relative to the wild-type containing an empty vector by a one-way ANOVA with Tukey’s multiple-comparison test. (G & H) S. aureus Newman wild-type, Δ*fdaB*, Δ*mntH*Δ*mntC*, and Δ*fdaB*Δ*mntH*Δ*mntC* were assessed for CP sensitivity in NRPMI containing 1 μM Zn, 1 μM Mn, and 1 μM Fe. Growth was assessed via the measurement of the absorbance at OD_600_ (H) at t = 6 h. *, *P* ≤ 0.05 relative to the wild-type strain at the CP concentration by a two-way ANOVA with Tukey’s multiple-comparison test. #, *P* ≤ 0.05 relative to the parental strain at the same CP concentration by a two-way ANOVA with Tukey’s multiple-comparison test. *n* ≥ 3. Error bars indicate the SEM.

While FbaA is a putatively Zn-dependent enzyme, CP can bind multiple first-row transition metals using two binding sites (26, 73–75). The first site (S1) can bind Mn, Zn, and Fe via six histidines, whereas the second site (S2) binds Zn, but not Mn or Fe, using three histidines and one aspartic acid (43, 44, 76, 77). To elucidate whether CP-imposed Zn limitation renders FdaB necessary for growth, wild-type CP and its ΔS1 and ΔS2 mutants, which have altered metal-binding properties, were leveraged. The ΔS1 mutant cannot bind Mn or Fe but retains the ability to bind Zn, whereas the ΔS2 variant binds all three metals. Surprisingly the Newman and USA300 JE2 *ΔfdaB* mutants have growth defects in the presence of ΔS2 but not in the presence of ΔS1 (Fig. 4A). This suggests that Zn limitation is not driving the necessity of FdaB for growth in the presence of CP. Given the unexpected nature of this finding, the ability of wild-type S. aureus and *ΔfdaB* to grow in a metal-defined medium, namely, NRPMI, which was lacking Mn, Zn, and Fe, was evaluated. Consistent with FdaB enabling S. aureus to survive metal starvation, the loss of FdaB ablated the growth of S. aureus Newman and USA300 JE2 in media to which Mn, Zn, and Fe were not added (Fig. 4B–F). The addition of Mn, but not Fe or Zn, reversed the growth defect (Fig. 4B, C, E, and F). Notably, differing from the results obtained using the TSB-based medium (Fig. 2B), *ΔfbaA* grew equivalent to the wild-type in NRPMI to which Mn, Zn, or Fe had been individually added back. This suggests that either all three metals must be present for the loss of FbaA to impact growth or that another component of the growth medium dictates the importance of FbaA. The ectopic expression of FdaB reversed the growth defect of the *ΔfdaB* mutant in Newman (Fig. 4D). Cumulatively, these results suggest that FdaB promotes resistance to Mn starvation. The loss of both S. aureus Mn transporters, MntABC and MntH, reduces intracellular Mn levels, compared to the wild-type (22). To further test the idea that FdaB is important for responding to Mn starvation, the impact that the loss of the staphylococcal Mn transporters has on *ΔfdaB* growth was investigated. Compared to Δ*fdaB* and Δ*mntC*Δ*mntH*, Δ*fdaB*Δ*mntC*Δ*mntH* is more sensitive to CP (Fig. 4G and H), with the double mutant having a growth defect, even in the absence of CP. This finding indicates that losing the ability to import Mn intensifies the impact of losing the metal-independent aldolase. While unexpected, cumulatively, these results suggest that FdaB is necessary for S. aureus to resist Mn limitation.

### FbaA activity is dependent on manganese.

**FIG 5:**
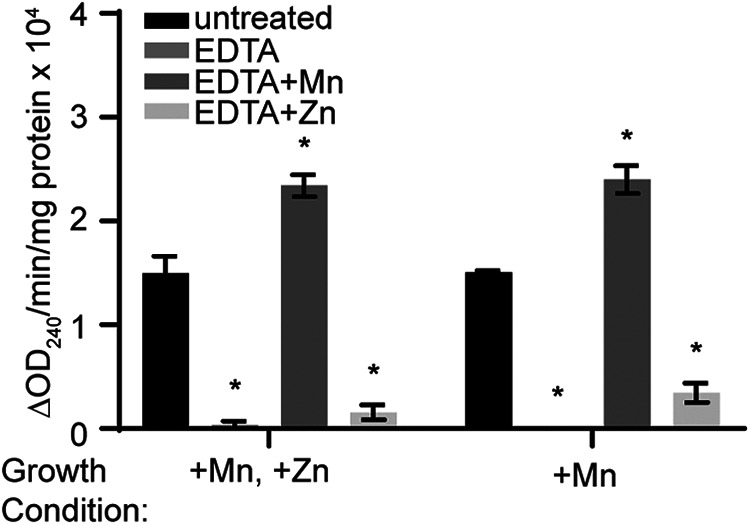
The activity of FbaA is Mn-dependent. S. aureus Newman Δ*fdaB* was grown in NRPMI containing 1 μM of the indicated metal. The aldolase activity was assessed. When indicated, the cell lysates were treated with EDTA, and Mn or Zn was added to the reaction. *, *P* ≤ 0.05 relative to the untreated by a two-way ANOVA with Tukey’s multiple-comparison test. *n* ≥ 3. Error bars indicate the SEM.

The unexpected finding that FdaB is necessary for the ability of S. aureus to resist Mn starvation called into question the Zn dependency of FbaA. To evaluate the metal dependency of FbaA, Δ*fdaB* was used to eliminate the activity of the metal-independent aldolase. As the Δ*fdaB* mutant grows poorly in NRPMI lacking Mn, aldolase activity was initially assessed following growth in medium supplemented with Mn or with Mn and Zn. Regardless of whether the medium contained Zn, similar levels of aldolase activity were observed in the Δ*fdaB* mutant (Fig. 5). To more directly test the metal dependency of FbaA, the cell lysates were treated with EDTA and then supplemented with either Mn or Zn. Treatment with EDTA eliminated the aldolase activity of the Δ*fdaB* mutant. The addition of excess Mn restored aldolase activity to comparable levels to those of untreated cell lysates (Fig. 5). In contrast, the addition of Zn resulted in a minimal increase in aldolase activity. Taken together, these observations suggest that FbaA requires Mn for activity.

### Both staphylococcal aldolases contribute to infection.

**FIG 6:**
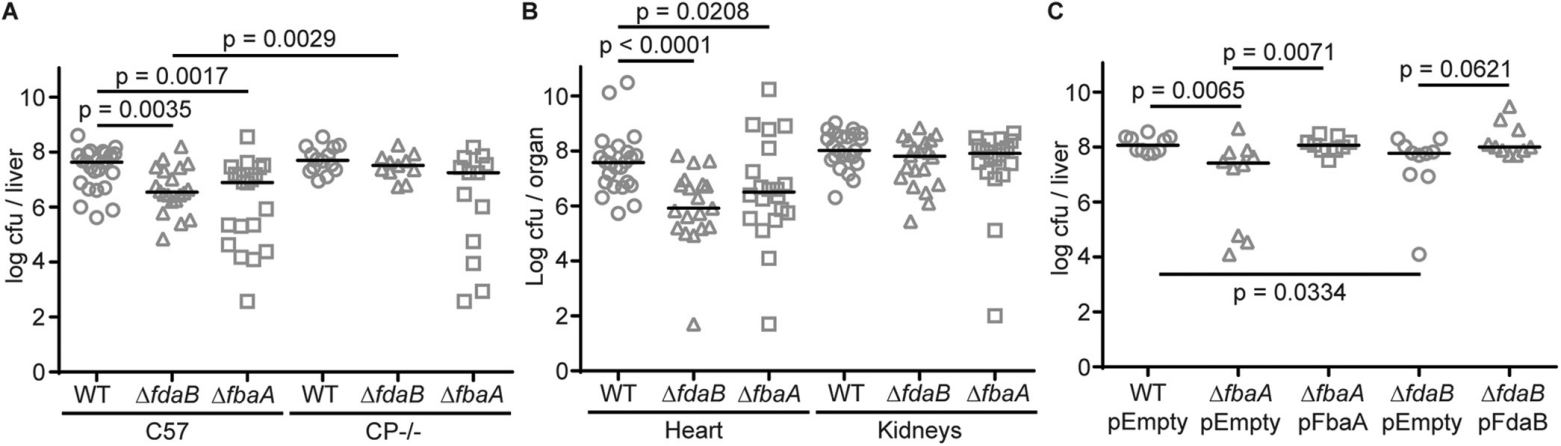
FdaB is necessary for the ability of S. aureus to cause infection. 9-week-old mice or C57BL/6J or CP-deficient mice (C57BL/6J S100A9^−/−^), were retro-orbitally infected with 1 × 10^7^ of S. aureus Newman wild-type, Δ*fbaA*, or Δ*fdaB*. After 4 days, the bacterial burdens in the (A) liver, (B) heart, and kidney were enumerated. (C) Newman wild-type, Δ*fbaA*, or Δ*fdaB* carrying either empty pKK30 (pEmpty), pKK30:*fbaA* (pFbaA), or pKK30:*fdaB* (pFdaB) were used to infect the mice, and after 4 days, the bacterial burdens were assessed. Statistical significance was evaluated via a Mann-Whitney U test. Specific *P* values for relevant comparisons are indicated. Bars indicate the median.

The current results suggest that FdaB enables S. aureus to resist Mn starvation in culture but that FbaA may be critical in metal-replete, glucose-containing environments. To determine the importance of the aldolase isozymes *in vivo*, a retro-orbital systemic model of staphylococcal infection was used. Initially, wild-type C57BL/6 mice were infected with wild-type S. aureus, *ΔfdaB*, and *ΔfbaA*. The bacterial burdens of mice infected with Δ*fdaB* and Δ*fbaA* were significantly decreased in the heart and liver, compared to those infected with wild-type S. aureus (Fig. 6A and B). These results suggest that both the metal-dependent and metal-independent aldolases are necessary for S. aureus to cause infection. Differing from the heart and liver, the loss of neither aldolase reduced the bacterial burdens in the kidney (Fig. 6B), suggesting that either aldolase is sufficient in this tissue. The expression of either FbaA or FdaB from a plasmid largely reversed the virulence defect of Δ*fbaA* or Δ*fdaB*, respectively (Fig. 6C). Next, CP-deficient mice, which fail to remove Mn from the liver during infection (9), were infected to determine whether the virulence defect of Δ*fdaB* is associated with an inability to cope with host-imposed Mn starvation. CP-deficient mice infected with wild-type S. aureus, Δ*fbaA*, or Δ*fdaB* had comparable bacterial burdens in the liver (Fig. 6A), indicating that FdaB is necessary to resist Mn starvation during infection. Cumulatively, these findings highlight the importance of both aldolases during infection and indicate that FdaB contributes to the ability of S. aureus to overcome Mn starvation.

## DISCUSSION

Nutrients, such as glycolic substrates and metals, are critical for pathogens during infection (2–5, 21, 25, 78–80). Glucose is the preferred carbon source for many invading pathogens, but it can also increase the cellular demand for Mn (13). This creates a challenge for S. aureus and for other pathogens, as glycolysis contributes to their ability to survive the assault by the immune system, but metal availability is also restricted at the sites of infections (46, 81). Therefore, to successfully cause infection, pathogens must balance rerouting metabolism to consume alternative energy sources, such as amino acids, with strategies that maximize their ability to retain glycolytic flux. The present work reveals that the metabolic regulator ArlRS, which has been implicated in the promotion of amino acid consumption (13), also regulates the expression of FdaB, a metal-independent aldolase that promotes resistance to host-imposed Mn starvation. This finding is unexpected, as the metal-dependent staphylococcal aldolase was predicted to depend on Zn, as do most metal-dependent versions of this enzyme (71, 82–87). Further investigation revealed that, differing from most previously characterized metal-dependent aldolases, which are Zn-dependent, FbaA utilizes Mn as a cofactor. Thus, the current work reveals aldolase as a target of nutritional immunity, a mechanism used by pathogens to preserve aldolase activity, and emphasizes the plasticity of enzyme metal specificity across organisms.

The surprising observation that FdaB is necessary to resist Mn starvation is not simply explained by the reduced expression of FbaA in Mn-deplete conditions, as the constitutive expression of FbaA does not reverse the growth defect of Δ*fdaB* in the presence of CP (Fig. 3A). Further investigation revealed that FbaA requires Mn for full activity (Fig. 5). Taken together, these observations suggest that S. aureus FbaA uses Mn, not Zn, as a cofactor. Whereas, to the best of our knowledge, S. aureus is the first pathogen to be identified to possess a Mn-dependent aldolase, Deinococcus radiodurans and Bacillus methanolicus also possess Mn-dependent class II fructose-1,6-bisphosphate aldolases (88, 89). Notably, all of these bacteria have intrinsically high intracellular Mn concentrations. Despite these prior findings and the similarity of class II aldolases to FbaA in S. aureus, class II aldolases are, by default, presumed to be Zn-dependent. This current work highlights the need to carefully evaluate metal dependency including consideration of the natural metal content of the host species, even for enzyme classes that have been extensively studied.

Aldolase is not the only staphylococcal glycolytic enzyme with two isoforms. PGM also has metal-dependent and metal-independent variants (72). Similar to the current observations, the metal-independent PGM isozyme, GpmA, contributes to resisting nutritional immunity (72). Notably, metal-independent glycolytic isozymes of PGM also promote resistance to Mn starvation in Salmonella (72). Differing from the staphylococcal PGMs, for which the metal-dependent isozyme appears to be dispensable in a systemic model of infection (72), both FdaB and FbaA are necessary for invasive disease in the heart and liver. This is despite the fact that, similar to PGM, both aldolases carry out the same chemical reaction. The classical explanation for the possession of two glycolytic enzymes is that one preferentially functions in glycolysis and the other preferentially functions in gluconeogenesis. This is one potential explanation for the nonredundancy of the staphylococcal aldolases, and this idea is supported by the observation that the loss of FbaA results in a growth defect in metal-replete media containing glucose. However, FdaB is capable of promoting S. aureus growth in glucose-containing media if Mn is limited. This suggests that environmental conditions, such as metal availability and regulatory cues, may drive the nonredundancy of the two staphylococcal aldolases, rather than specific roles in glycolysis and gluconeogenesis. Alternatively, metal-dependent aldolases from Neisseria meningitidis, Mycoplasma hyopneumoniae, and Francisella novicida have been associated with moonlighting functions, such as acting as transcriptional regulators and adhesins (90–92). While both aldolases are necessary for the infection of the liver and heart, either is sufficient in the kidneys. This suggests that in some tissues, they serve the same purpose or that despite the kidney being a Mn-restricted environment, the need for aldolase activity is reduced to a point that residual activity from FbaA is sufficient. Alternatively, it is possible that aldolase activity is dispensable in the kidney. Regardless of the rationale for why FbaA contributes to S. aureus infection, the current results establish an important role for the metal-independent isozyme FdaB in resisting Mn starvation during infection.

There are two different classes of aldolases: class-I and class-II, where class-I are metal-independent and class-II are metal-dependent, and bacteria can possess one or more of these enzymes (90). When bacteria express a single aldolase, it is most commonly metal-dependent, belonging to class II (90). However, certain bacteria, including S. aureus, Escherichia coli, and Mycobacterium tuberculosis express two aldolases, with one belonging to class I and the other to class II (93–96). Similar to that of S. aureus, the metal-independent aldolase of E. coli has been suggested to favor gluconeogenesis (70). However, the current work raises the possibility that possessing a second metal-independent aldolase may also facilitate the ability of bacteria to survive metal starvation or potentially other stresses. Although, in the case of E. coli, it seems more likely that the metal-independent aldolase would promote resistance to Zn limitation, as the metal-dependent isozyme relies on that metal for function (97). Interestingly, Bacillus methanolicus possesses two apparently metal-dependent aldolases (98), with the molecular rationale remaining unknown. The observation that not all aldolases are Zn-dependent raises the possibility that the second aldolase in this species may also promote resistance to metal limitation if the two isozymes have differing metal specificities.

The current observations suggest that ArlRS also aids in the preservation of glycolytic flux by modulating the expression of one of the staphylococcal aldolases. While ArlRS is not necessary to induce the expression of *fdaB* in response to CP, the loss of this TCS does ablate FdaB expression (Fig. 2A). This observation suggests the existence of additional regulators that modulate FdaB expression in response to metal limitation. It is tempting to speculate that the upregulation of FdaB by ArlRS might enhance gluconeogenesis and thereby allow for the production of essential biosynthetic precursors that lay upstream of aldolase. This role for ArlRS would also be consistent with its apparent ability to sense the accumulation of metabolites from the latter half of the glycolytic pathway and with the reported role of FdaB in gluconeogenesis (14, 99). In addition to withholding essential metals, the host has other defenses that can disrupt glycolytic flux, including the production of itaconate, which can inhibit FbaA, among other enzymes (100). Given the importance of aldolase activity to glycolysis and the multiple ways by which the host can target this pathway, it is perhaps unsurprising that multiple regulators can control the expression of *fdaB*. While multiple host defenses can target FbaA, the observation that the loss of CP reverses the virulence defect of Δ*fdaB* suggests that this alternative isozyme is important for resting nutritional immunity.

Adaptation to host-imposed environmental challenges is critical for bacterial survival. The expression of two different aldolases with different biochemical properties supports the importance of the redundancy of certain enzymes that are critical in coping with the different stresses that pathogens encounter during infection. It also highlights this pathogen’s remarkable metabolic plasticity and ability to adapt to hostile host milieu.

## MATERIALS AND METHODS

### Ethics statement.

All experiments involving animals were approved by the Institutional Animal Care and Use Committee of the University of Illinois at Urbana-Champaign (IACUC license number 15059) and were performed according to NIH guidelines, the Animal Welfare Act, and U.S. federal law.

### Strains and growth conditions.

S. aureus strains were grown at 37°C in tryptic soy broth with glucose (TSB) on a roller drum or on tryptic soy agar (TSA) plates for the performance of routine culturing or for genetic manipulation. E. coli strains were routinely cultivated at 37°C in Luria broth (LB) with shaking or on Luria agar plates. As needed for plasmid maintenance in E. coli and S. aureus, 100 μg/mL of ampicillin and 10 μg/mL of chloramphenicol were added to the growth media, respectively. Both bacterial species were stored at −80°C in a growth medium that contained 30% glycerol.

S. aureus Newman or USA300 (JE2) and derivatives were used for all of the experiments. For the overnight cultures, the bacteria were grown in 5 mL of either tryptic soy broth with glucose (TSB) or Chelex-treated RPMI plus 1% Casamino Acids (NRPMI) supplemented with 1 mM MgCl_2_, 100 μM CaCl_2_, and 1 μM FeCl_2_ in 15 mL conical tubes at 37°C on a roller drum (13, 23). 10 μg/mL of chloramphenicol was added as need for plasmid maintenance. The hemolytic activity of all staphylococcal strains was confirmed via plating on blood agar plates. The strains used in this study are listed in Table 1. The *fbaA::erm* and *fdaB::erm* alleles were obtained from the Nebraska Transposon Mutant Library (NTML) and were introduced into S. aureus Newman and USA300 JE2 via phage transduction. Plasmids in the pOS1plgt background were constructed with the indicated primers (Table 2) via restriction cloning, whereas those in the pKK30 background were constructed via Gibson assembly.

**TABLE 1:** Staphylococcus aureus strains and plasmids used in this study

Bacterial strains	Genotype	Source
Newman WT	Wild-type methicillin sensitive strain	105
Newman Δ*fbaA*	*fbaA::erm*	This study
Newman Δ*fdaB*	*fdaB::erm*	This study
USA300 JE2	Wild-type methicillin resistant strain	NTML (ID: NR-46543)
USA300 JE2 Δ*fbaA*	*fbaA::erm*	NTML (ID: NR-47019)
USA300 JE2 Δ*fdaB*	*fdaB::erm*	NTML (ID: NR-46831)
Newman Δ*arlRS*	Δ*arlRS*	13
Newman Δ*fdaB* Δ*arlRS*	*fdaB::erm* Δ*arlRS*	This study
Newman Δ*mntC*Δ*mntH*	Δ*mntC*Δ*mntH*	13
Newman Δ*fdaB*Δ*mntC*Δ*mntH*	*fdaB::erm* Δ*mntC*Δ*mntH*	This study
pOS1 plgt empty	pOS1 plgt without an insert	48
pOS1 plgt:*fbaA*	pOS1 plgt with *fbaA* under the control of the lgt promoter	This study
pOS1 plgt:*fdaB*	pOS1 plgt with *fdaB* under the control of the lgt promoter	This study
pKK30 empty	pKK30 vector without an insert	106
pKK30:*fbaA*	pKK30 with *fbaA* under the control of the native promoter	This study
pKK30:*fdaB*	pKK30 with *fdaB* under the control of the native promoter	This study

**TABLE 2:** Primers used in this study

Name	Sequence
2503 F NdeI (for *fbaA* complementation)	AGTCCATATGAATAAAGAGCAATTAGAAAAAATG
2503 R BamHI (for *fbaA* complementation)	AGTCGGATCCTTAGTTTTTGTTTACAGAT
2029 F NdeI (for *fdaB* complementation)	AGTCCATATGCCTTTAGTTTCAATGAAAGAAATG
2029 R BamHI (for *fdaB* complementation)	AGTCGGATCCTTATTTAGCGCGGTTAGAAGTAC
2503 F (for *fbaA* expression analysis)	GGATCGCGAAGTAGAAAGCA
2503 R (for *fbaA* expression analysis)	AATGTGACGTTCGTTTGCAC
2029 F (for *fdaB* expression analysis)	TTCAGCAAAAGCAGTTCGTG
2029 R (for *fdaB* expression analysis)	TAGCGCGGTTAGAAGTACCG
*fbaA* pKK30 F (for *fbaA* complementation)	TGCTTGTAATTCATGATTCGTCTACTTATAAAATATTGTAATTAATGACTACATATTATG
*fbaA* pKK30 R (for *fbaA* complementation)	TCATATATCAAGCAAAGTGACAGGCGATGCTTATTTAGCGCGGTTAGAAG
*fdaB* pKK30 F (for *fdaB* complementation)	TTTTGCTTGTAATTCATGATTCGTTTAATTAAACTGACAATTATTTTTCACATTTTATAC
*fdaB* pKK30 R (for *fdaB* complementation)	TCATATATCAAGCAAAGTGACAGGCGATGCTTAGTTTTTGTTTACAGATGCGTC

### Transcriptome profiling.

S. aureus Newman wild-type and Δ*arlRS* were grown in TSB with glucose overnight. Then, the cultures were diluted 1:100 into 96-well round-bottom plates containing 100 μL of growth medium (38% TSB [with or without glucose] and 62% calprotectin buffer [20 mM Tris pH 7.5, 100 mM NaCl, 3 mM CaCl2, 10 mM β-mercaptoethanol]). The growth medium was supplemented with 1 μM MnCl_2_ and 1 μM ZnSO_4_. Bacteria were harvested during log-phase growth (OD_600_ of approximately 0.1), and an equal volume of ice-cold 1:1 acetone-ethanol was then added to the cultures before freezing at −80°C until RNA extraction. RNA was extracted, and cDNA was generated as previously described (101–103). Purified RNA was submitted for RNA-seq preparation and sequencing at the Roy J. Carver Biotechnology Center (CBC) at the University of Illinois Urbana-Champaign.

### Expression analysis.

To assess the expression of *fbaA* and *fdaB*, S. aureus Newman was grown in TSB with glucose overnight. Then, the cultures were diluted 1:100 into 96-well round-bottom plates containing 100 μL of growth medium (38% TSB with glucose and 62% calprotectin buffer [20 mM Tris pH 7.5, 100 mM NaCl, 3 mM CaCl2, 10 mM β-mercaptoethanol]) in the presence and absence of 240 μg/mL of CP. The growth medium was supplemented with 1 μM MnCl_2_ and 1 μM ZnSO_4_. Bacteria were harvested, RNA was extracted, and cDNA was prepared as indicated above for transcriptome profiling. Gene expression was assessed via quantitative reverse transcription-PCR (qRT-PCR), using the indicated primers (Table 2), with 16S being used as a normalizing control.

### Calprotectin growth assays.

CP assays were largely performed as described previously (13, 27, 43). Overnight cultures grown in TSB with glucose were diluted 1:50 into 5 mL of fresh medium and were then incubated for 1 h or, if the strain contained a plasmid, 2 h at 37°C on a roller drum. The cultures were then back-diluted 1:100 in 96-well round-bottom plates containing 100 μL of growth medium (38% 3 × NRPMI and 62% calprotectin buffer [20 mM Tris pH 7.5, 100 mM NaCl, 3 mM CaCl_2_]) in the presence of various concentrations of CP. The growth medium was supplemented with 1 μM MnCl_2_, 1 μM FeCl_2_, and 1 μM ZnSO_4_. For all assays, the bacteria were incubated with orbital shaking (180 rpm) at 37°C, and growth was measured by assessing the optical density (OD_600_) every 1 to 2 h. Prior to the measurement of the optical density, the 96-well plates were vortexed.

### Metal starvation growth assays.

For the growth assays using Chelex-treated medium to impose metal limitation, overnight cultures grown in NRPMI that contained 1 mM MgCl_2_ and 100 μM CaCl_2_, were diluted 1:10 in fresh medium that lacked metals before being further diluted 1:100 in 96-well round-bottom plates containing NRPMI supplemented with 1 mM MgCl_2_ and 100 μM CaCl_2_. As specified, 1 μM MnCl_2_, 1 μM ZnSO_4_, and 1 μM FeCl_2_ were also added. The bacteria were incubated with orbital shaking (180 rpm) at 37°C, and growth was measured by assessing the optical density (OD_600_) every 1 to 2 h. Prior to the measurement of the optical density, the 96-well plates were vortexed.

### Aldolase activity assays.

Overnight cultures grown in NRPMI containing 1 mM MgCl_2_, 100 μM CaCl_2_, and 1 μM FeCl_2_ were diluted 1:10 in fresh medium before being further diluted 1:100 in 96-well round-bottom plates containing NRPMI supplemented with 1 mM MgCl_2_, 100 μM CaCl_2_, and 1 μM FeCl_2_. Additionally, 1 μM MnCl_2_ and/or 1 μM ZnSO_4_ were added as specified. Bacteria were harvested during logarithmic-phase growth (*t* = 6 h), with approximately 8 mL of cell culture per sample being harvested via centrifugation. The bacterial pellets were washed with 10 mL of 50 mM Tris-HCl (pH 7.5), before resuspension in 1 mL of this buffer. Prior to assaying aldolase activity, the cells were homogenized twice in a FastPrep-24 Beadbeater at 6 m/s for 45 s cycles with 5 min of incubation on ice in between. The cell lysates were centrifuged at 4°C in a microcentrifuge at 14,000 × *g* for 10 min. The supernatants were collected and used for the aldolase activity assay, which was performed as described by Zhang, et al., with a few modifications (88). Briefly, aldolase activity was determined by mixing untreated or EDTA-treated supernatants, 2 mM hydrazine, and 2.4 mM fructose-1,6-bisphosphate in 50 mM Tris-HCl (pH 7.5). Glyceraldehyde-3-phosphate produced from fructose-1,6-bisphosphate reacts with hydrazine to form an aldehyde-hydrazone, the production of which was measured via the tracking of the absorbance at 240 nm after 1 h of incubation at 25°C. Supernatants treated with 0.67 nM EDTA were incubated for 10 min at 25°C prior to their use for the aldolase activity assay. When indicated, 1 mM MnCl_2_ or 1 mM ZnSO_4_ was added to the reaction. For normalization, the total protein was determined using a BCA assay. Activity was defined as the change of the absorbance at 240 nm per minute per mg of total protein.

### Animal experiments.

All animal infections were performed as previously described (13, 48, 104). 9-week-old female C57BL/6 or S100A9^−/−^ mice were retro-orbitally infected with approximately 1 × 10^7^ CFU suspended in 100 μL of sterile PBS. The infection was allowed to proceed for 4 days, after which the mice were sacrificed. The liver, heart, and kidneys were collected. These organs were homogenized, and the bacterial burdens were determined via the plating of serial dilutions.

### Data availability.

Transcriptional profiling data were deposited in the NCBI Gene Expression Omnibus (GEO) repository (accession number: GSE202268).
